# Design of Self-Oscillating Gels and Application to Biomimetic Actuators

**DOI:** 10.3390/s100301810

**Published:** 2010-03-05

**Authors:** Ryo Yoshida

**Affiliations:** Department of Materials Engineering, Graduate School of Engineering, The University of Tokyo, 7-3-1 Hongo, Bunkyo-ku, Tokyo 113-8656, Japan; E-Mail: ryo@cross.t.u-tokyo.ac.jp; Tel.: +81-3-5841-7112; Fax: +81-3-5841-7112

**Keywords:** polymer gel, actuator, self-oscillation, biomimetics

## Abstract

As a novel biomimetic polymer, we have developed polymer gels with an autonomous self-oscillating function. This was achieved by utilizing oscillating chemical reactions, called the Belousov-Zhabotinsky (BZ) reaction, which is recognized as a chemical model for understanding several autonomous phenomena in biological systems. Under the coexistence of the reactants, the polymer gel undergoes spontaneous swelling-deswelling changes without any on-off switching by external stimuli. In this review, our recent studies on the self-oscillating polymer gels and application to biomimetic actuators are summarized.

## 1. Introduction

Over about the last two or three decades, many kinds of stimuli-responsive polymer gels, which respond to changes in their surroundings such as temperature, pH, and supply of electric field, *etc.*, have been developed. They have attracted much attention as smart (or biomimetic) materials, and several applications to actuator (artificial muscle), biosensor, drug delivery systems, purification or separation systems, tissue engineering, *etc.* are extensively studied [1].

Several mechanical devices using gels were devised and demonstrated in the late 80s and early 90s when gels first began to attract attention as a functional material [2]. For example, devices driven by a change in temperature or giving an electric field [3] (artificial muscles or robot hands that lift or grasp something, artificial fish that swim with repetitive bending motions, artificial looper that walks on rails [4], *etc.*) attracted much attention. In addition, several chemomechanical gels, e.g., “biochemo-mechanical” gel that contracts when a substrate for enzymatic reaction is added in outside solution, were demonstrated [5].

Recently, electrically stimulated systems using polyelectrolyte gels, organogels [6], gels consisting of carbon nanotube and ionic liquid [7], *etc.* have become popular and some materials have already come to near practical use. For example, the ion conductive polymer actuator obtained by plating a golden electrode on both sides of a perfluoro carboxylic acid film causes bending motion and biomimetic motion by electric field addition. By using the gels, hobby products such as an artificial fish (has already beencommercialized), medical operation devices (catheter, *etc.*), *etc.* are devised [8] also. In addition, gel use as actuating systems driven by a magnetic field was studied by Zrinyi *et al.* [9]. They prepared PVA gel containing magnetite (Fe_3_O_4_) particles (ferrogel) and demonstrated that the gel showed dynamic motion in response to a magnetic field.

As one of the characteristic behaviors in living systems, autonomous oscillation - that is, spontaneous changes with temporal periodicity (called “temporal structure”) such as heartbeat, brain waves, pulsatile secretion of hormone, cell cycle, biorhythm - can be exemplified. From the standpoint of biomimetics, several stimuli-responsive polymer systems have been studied, but the polymer systems undergoing self-oscillation under constant condition without any on-off switching of external stimuli are still undeveloped. If such autonomous polymer systems like a living organism can be realized by using completely synthetic polymers, then unprecedented biomimetic materials will be created.

We attempted to develop a novel gel that provides mechanical oscillation by itself without external control in a completely closed solution. We succeeded in developing such a self-oscillating polymer and gels by incorporating oscillating chemical reactions in polymer network, *i.e.,* by constructing a built-in circuit of energy conversion cycle producing mechanical oscillation within the polymer network itself. Under the coexistence of the reactants in a closed and constant-conditioned solution, the polymer undergoes spontaneous cyclic soluble-insoluble changes or swelling-deswelling changes (in the case of gel) without any on-off switching by external stimuli, thus differently from conventional stimuli-responsive gels. Since it was first reported in 1996 as “self-oscillating gel” [10], we have been systematically studying the self-oscillating polymer and gel as well as their applications to biomimetic or smart materials [11–13]. In this review, these recent progress on the self-oscillating polymer gels and the design of autonomic and biomimetic actuators are summarized.

## 2. Design of Self-Oscillating Gel

In order to realize the autonomous polymer system by tailor-made molecular design, we focused on the Belousov-Zhabotinsky (BZ) reaction [14,15], which is well-known for exhibiting temporal and spatiotemporal oscillating phenomena. The BZ reaction is often analogically compared with the TCA cycle (Krebs cycle), which is a key metabolic process taking place in the living body. The overall process of the BZ reaction is the oxidation of an organic substrate, such as malonic acid (MA) or citric acid, by an oxidizing agent (bromate ion), in the presence of a strong acid and a metal catalyst. In the course of the reaction, the catalyst undergoes spontaneous redox oscillation. When the solution is homogeneously stirred, the color of the solution periodically changes, like a neon sign, based on the redox changes of the metal catalyst. When the solution is placed as a thin film in stationary conditions, concentric or spiral wave patterns develop in the solution. The wave of oxidized state propagating in the medium at a constant speed is called a “chemical wave”.

**Figure 1.**
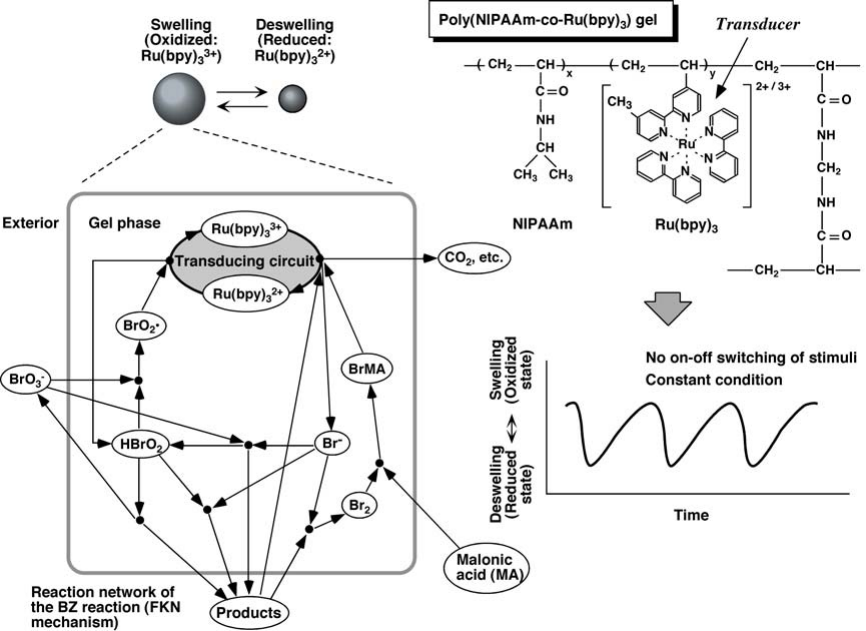
Mechanism of self-oscillation for poly(NIPAAm-co-Ru(bpy)_3_^2+^) gel coupled with the Belousov-Zhabotinsky reaction.

It was attempted to convert the chemical oscillation of the BZ reaction to the mechanical changes of gels and generate an autonomic swelling-deswelling oscillation under nonoscillatory outer conditions. A copolymer gel, which consists of NIPAAm and ruthenium tris (2,2′-bipyridine) (Ru(bpy)_3_^2+^) was prepared. Ru(bpy)_3_^2+^, acting as a catalyst for the BZ reaction, is pendent to the polymer chains of NIPAAm (Figure 1). The poly(NIPAAm-co-Ru(bpy)_3_^2+^) gel has a phase transition temperature because of the themosensitive constituent NIPAAm. The oxidation of the Ru(bpy)_3_^2+^ moiety caused not only an increase in the degree of swelling of the gel, but also a rise in the transition temperature. These characteristics may be interpreted by considering an increase in hydrophilicity of the polymer chains due to the oxidation of Ru(II) to Ru(III) in the Ru(bpy)_3_ moiety. As a result, it is expected that the gel undergoes a cyclic swelling-deswelling alteration when the Ru(bpy)_3_ moiety is periodically oxidized and reduced under constant temperature. When the gel is immersed in an aqueous solution containing the substrates of the BZ reaction (MA, acid, and oxidant) except for the catalyst, the substrates penetrates into the polymer network and the BZ reaction occurs in the gel. Consequently, periodical redox changes induced by the BZ reaction produce periodical swelling-deswelling changes of the gel (Figure 1).

## 3. Self-Oscillating Motion of Miniature Bulk Gel: Homogeneous Swelling-Deswelling Oscillation

**Figure 2.**
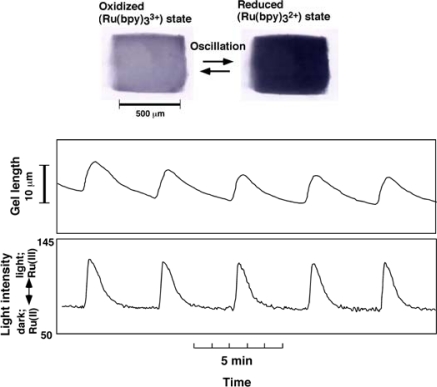
Periodic redox changes of the miniature cubic poly(NIPAAm-co-Ru(bpy)_3_^2+^) gel (lower) and the swelling-deswelling oscillation (upper) at 20 °C. Color changes of the gel accompanied by redox oscillations (orange: reduced state, light green: the oxidized state) were converted to 8-bit grayscale changes (dark: reduced, light: oxidized) by image processing. Transmitted light intensity is expressed as an 8-bit grayscale value. Outer solution: [MA] = 62.5mM; [NaBrO_3_] = 84mM; [HNO_3_] = 0.6M (Reference [16]).

Figure 2 shows the oscillating behavior observed under a microscope for the miniature cubic poly (NIPAAm-co-Ru(bpy)_3_^2+^) gel (each with a length of about 0.5 mm). In miniature gels sufficiently smaller than the wavelength of the chemical wave (typically several mm), the redox change of the ruthenium catalyst can be seen to occur homogeneously without pattern formation [16]. Due to the redox oscillation of the immobilized Ru(bpy)_3_^2+^, mechanical swelling-deswelling oscillation of the gel autonomously occurs with the same period as for the redox oscillation. The volume change is isotropic and the gel beats as a whole, like a heart muscle cell. The chemical and mechanical oscillations are synchronized without a phase difference (*i.e.,* the gel exhibits swelling during the oxidized state and deswelling during the reduced state).

Typically, the oscillation period increases with a decrease in the initial concentration of substrates. The swelling-deswelling amplitude of the gel increases with an increase in the period and amplitude of the redox changes. Therefore the swelling-deswelling amplitude of the gel is controllable by changing the initial concentration of substrates. As an inherent behavior of the BZ reaction, the abrupt transition from steady state (non-oscillating state) to oscillating state occurs with a change in controlling parameters such as chemical composition, light, *etc.* By utilizing this characteristic, reversible on-off regulation of self-beating triggered by addition and removal of MA was successfully achieved [17]. In addition, as the gel is thermosensitive due to the NIPAAm component, the beating rhythm can be also controlled by temperature [18].

## 4. Worm-Like Peristaltic Motion of Gel

**Figure 3.**
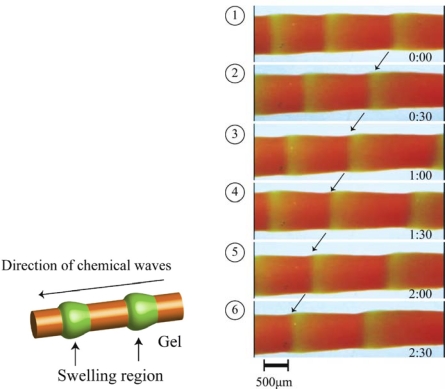
Time course of peristaltic motion of poly(NIPAAm-co-Ru(bpy)_3_^2+^-co-AMPS) gel in a solution of the BZ substrates (MA, NaBrO_3_ and HNO_3_, 18 °C). The green and orange colors correspond to the oxidized and reduced states of the Ru moiety in the gel, respectively (Reference [22]).

When the gel size is larger than the chemical wavelength, the chemical wave propagated in the gel is coupled with diffusion of intermediates [19–21], resulting in creation of peristaltic motion of the gel. Figure 3 shows the cylindrical gel, which is immersed in an aqueous solution containing the three reactants of the BZ reaction. The chemical waves propagate in the gel at a constant speed in the direction of the gel length. Considering the orange (Ru(II)) and green (Ru(III)) zones represent simply the shrunken and swollen parts, respectively, the locally swollen and shrunken parts move with the chemical wave, like the peristaltic motion of living worms (Figure 3) [22]. The tensile force of the cylindrical gel with oscillation was also measured [23,24].

It is well known that the period of oscillation is affected by light illumination for the Ru(bpy)_3_^2+^-catalyzed BZ reaction [25]. Therefore, it is possible to intentionally make a pacemaker with a desired period (or wavelength) by local illumination of laser beam to the gel, or change the period (or wavelength) by local illumination to a pacemaker that already exists in the gel [26]. Chemical and optical control of the worm-like peristaltic motion of a structural colored porous gel were demonstrated [27–29].

## 5. Design of Biomimetic Actuator Using Self-Oscillating Gel

### 5.1. Self-Walking Gel

Further, a novel biomimetic walking-gel actuator made of self-oscillating gel was successfully developed [30]. To produce directional movement of the gel, asymmetrical swelling-deswelling is desired. For these purposes, as a third component, hydrophilic 2-acrylamido-2-methylpropanesulfonic acid (AMPS) was copolymerized into the polymer to lubricate the gel and to cause anisotropic contraction. During polymerization, the monomer solution faces two different plate surfaces; a hydrophilic glass surface and a hydrophobic Teflon surface. Since Ru(bpy)_3_^2+^ monomer is hydrophobic, it easily migrates to the Teflon surface side. As a result, a non-uniform distribution along the height is formed by the components, and the resulting gel has a gradient distribution for the content of each component in the polymer network.

**Figure 4.**
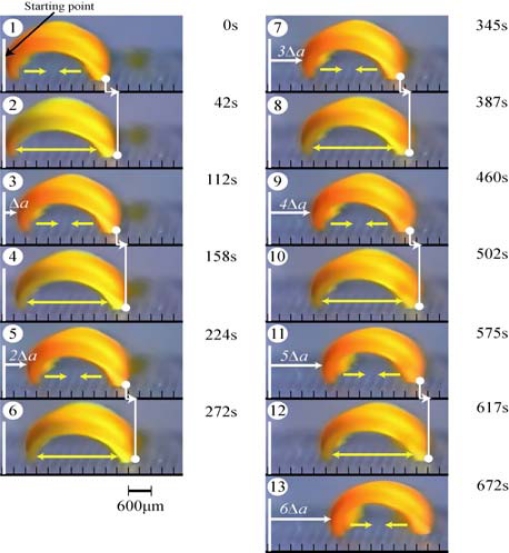
Time course of self-walking motion of the gel actuator in a solution of the BZ substrates (MA, NaBrO_3_ and HNO_3_, 18 °C). During stretching, the front edge can slide forward on the base, but the rear edge is prevented from sliding backwards. Oppositely, during bending, the front edge is prevented from sliding backwards while the rear edge can slide forward. This action is repeated, and as a result, the gel walks forward (Reference [30]).

In order to convert the bending and stretching changes to one-directional motion, we employed a ratchet mechanism. A ratchet base with an asymmetrical surface structure was fabricated. On the ratchet base, the gel repeatedly bends and stretches autonomously resulting in the forward motion of the gel, while sliding backwards is prevented by the teeth of the ratchet. Figure 4 shows successive profiles of the “self-walking” motion of the gel like a looper in the BZ substrate solution under constant temperature. The walking velocity of the gel actuator was approximately 170 μm/min. Since the oscillating period and the propagating velocity of the chemical wave change with concentration of substrates in the outer solution, the walking velocity of the gel can be controlled. By using the gel with gradient structure, other type of actuator which generates a pendulum motion is also realized [31].

### 5.2. Mass Transport Surface Utilizing Peristaltic Motion of Gel

**Figure 5.**
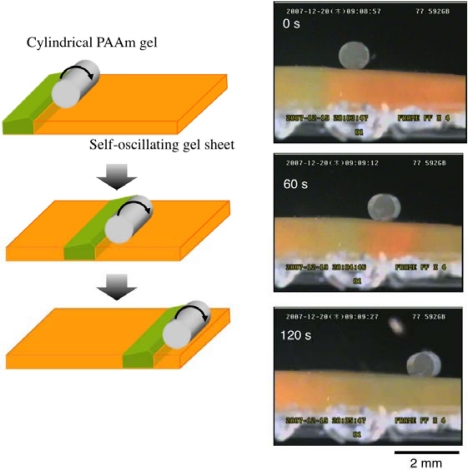
Schematic illustration of mass transport on the peristaltic surface (left) and observed transport of cylindrical PAAm gel on the poly (NIPAAm-co-Ru(bpy)_3_^2+^-co-AMPS) gel sheet in a solution of the BZ substrates (MA, NaBrO_3_ and HNO_3_) (right) (Reference[32]).

It was also attempted to transport an object by utilizing the peristaltic motion of poly (NIPAAm-*co*-Ru(bpy)_3_-*co*-AMPS) gels. Here AMPS was copolymerized to increase the swelling-deswelling amplitude. As a model object, a cylindrical poly (acrylamide) (PAAm) gel was put on the gel surface. It was observed that the PAAm gel was transported on the gel surface with the propagation of the chemical wave as it rolled [22,32,33] (Figure 5). We have proposed a model to describe the mass transport phenomena based on the Hertz contact theory, and the relation between the transportability and the peristaltic motion was investigated. The functional gel surface generating autonomous and periodic peristaltic motion has a potential for several new applications such as a conveyer to transport soft materials, a formation process for ordered structures of micro- and/or nanomaterials, a self-cleaning surface, *etc*.

### 5.3. Ciliary Motion Actuator (Artificial Cilia)

One of the promising fields of the MEMS is micro actuator array or distributed actuator systems. The actuators, which have a very simple actuation motion such as up and down motion, are arranged in an array form. If their motions are random, no work is extracted from this array. However, by controlling them to operate in a certain order, they can generate work as a system. A typical example of this kind of actuation array is a ciliary motion micro actuator array. There have been many reports on this system. Although various actuation principles have been proposed, all previous work is based on the concept that the motion of actuators is controlled by external signals. If a self-oscillating gel plate with a micro projection structure array on top was realized, it would be expected that the chemical wave propagation would create dynamic rhythmic motion of the structure array. This proposed structure could exhibit spontaneous dynamic propagating oscillation producing a ciliary motion array.

**Figure 6.**
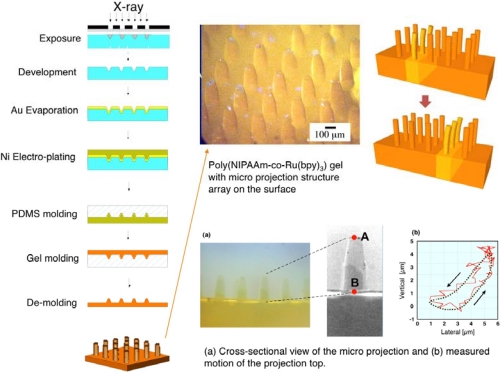
Fabrication of ciliary motion actuator (artificial cilia) using self-oscillating gel (Reference [34]).

A gel plate with micro projection array was fabricated by molding (Figure 6) [34,35]. First, moving mask deep-X-ray lithography was utilized to fabricate a PMMA plate with a truncated conical shape microstructure array. This step was followed by evaporation of a Au seed layer and subsequent electroplating of nickel to form the metal mold structure. Then, a PDMS mold structure was duplicated from the Ni structure and utilized for gel molding. The formation of gel was carried out by vacuum injection molding. A structure with a height of 300 μm and bottom diameter of 100 μm was successfully fabricated by the described process. The propagation of chemical reaction wave and dynamic rhythmic motion of the micro projection array were confirmed by chemical wave observation and displacement measurements. Figure 6 shows the measured lateral and vertical movements and the motion trajectory of the projection top. Motion of the top with 5 μm range in both lateral and vertical directions, and elliptical motion of the projection top were observed.

The feasibility of the new concept of the ciliary motion actuator made of self-oscillating polymer gel was successfully confirmed. The actuator may serve as a micro-conveyer to transport micro- or nano-particles on the surface. Currently, we are trying to develop a chemical robot, which is unlike a conventional electrically powered robot, by coupling with a PDMS membrane [36].

### 5.4. Control of Chemical Wave Propagation in Self-Oscillating Gel Array

**Figure 7.**
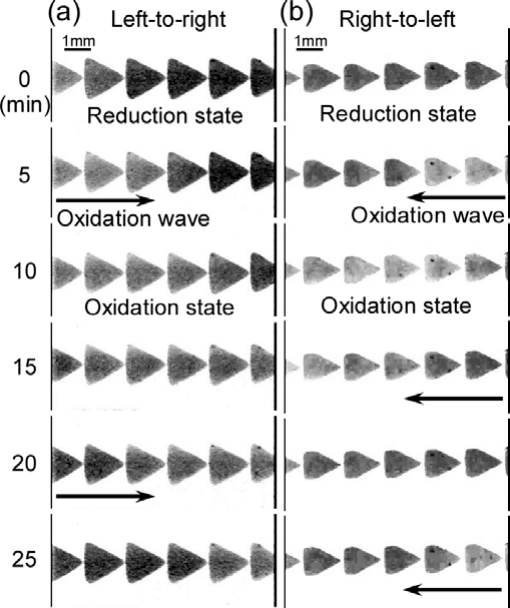
Propagating behavior of the chemical wave on the (a) triangular gel array and (b) pentagonal gel array (Reference [37]).

A chemomechanical actuator utilizing a reaction-diffusion wave across gap junction was constructed toward a novel mircoconveyer by micropatterned self-oscillating gel array [37]. Unidirectional propagation of the chemical wave, the BZ reaction, was induced on gel arrays. In the case of using a triangle-shaped gel as an element of the array, the chemical wave propagated from the corner point of the triangular gel to the plane side of the other gel (C-to-P) across the gap junction, whereas it propagated from the plane side to the corner side (P-to-C) in the case of the pentagonal gel array (Figure 7). Numerical analysis based on theoretical modeling was done to understand the mechanism of unidirectional propagation in triangular and pentagonal gel arrays. By fabricating different shapes of gel arrays, control of the direction is possible. The swelling and deswelling changes of the gels followed the unidirectional propagation of the chemical wave. Applications in novel microconveyers is expected.

## 6. Conclusions

**Figure 8.**
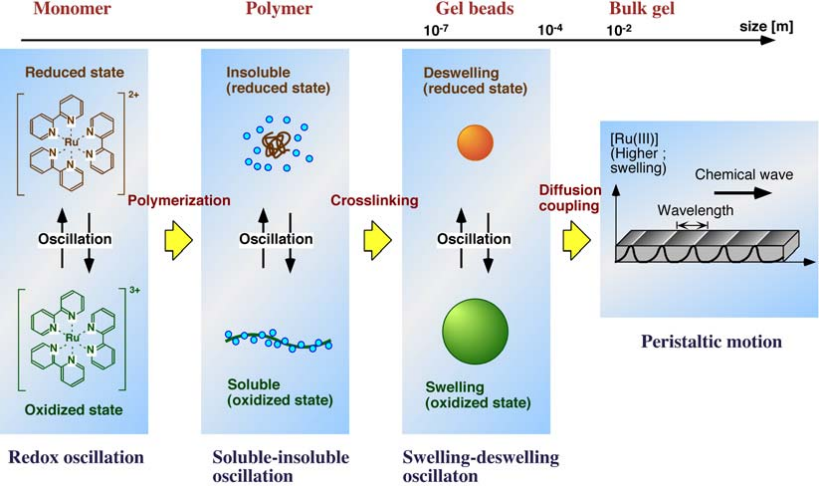
Synchronization in self-oscillating gel over the range from microscopic to macroscopic level.

In self-oscillating gel, redox changes of Ru(bpy)_3_^2+^ catalyst are converted to conformational changes of polymer chain by polymerization. These periodic changes of linear and uncrosslinked polymer chains can be easily observed as cyclic transparent and opaque changes for the polymer solution with color changes due to the redox oscillation of the catalyst [38]. The conformational changes are amplified to macroscopic swelling-deswelling changes of the polymer network by crosslinking. Further, when the gel size is larger than the chemical wavelength, the chemical wave propagates in the gel by coupling with diffusion. In this case, peristaltic motion of the gel is created. In this manner, a synchronization process exists from the microscopic to the macroscopic level in the self-oscillating gel (Figure 8).

These self-oscillating gels may be useful in a number of important applications to autonomous actuator as mentioned in this review. Furthermore, in order to realize nano-actuator exhibiting autonomous oscillation (nano-oscillator), the linear polymer chain and the submicrometer-sized gel beads were actually prepared [39–43]. The self-oscillating behaviors were analyzed, and the crosslinking effects on the inter- and intrapolymer interaction under the micro-environment have been discussed. By grafting the polymers or arraying the gel beads on the surface of substrates, we have attempted to design self-oscillating surfaces as nano-conveyers to transport microparticles, *etc.* with the spontaneous propagation of chemical waves [44,45]. For applications in biomaterials, it is necessary to cause the self-oscillation under biological conditions without using non-biorelated BZ substrates. We attempted to introduce a pH-control site and an oxidant-supplying site into the polymer [46–49]. By using the polymer, self-oscillation was achieved only in the presence of biorelated organic acid. Applications in biomimetic and autonomous micro- or nano-actuators are expected.
